# Supplementary material for: Vocal learning promotes patterned inhibitory connectivity
Source: Nat Commun. 2017 Dec 13;8:2105. doi: 10.1038/s41467-017-01914-5 (PMC5727387; doi:10.1038/s41467-017-01914-5)
Supplement: Supplementary file 1 — Supplementary Information [file 41467_2017_1914_MOESM1_ESM.pdf]

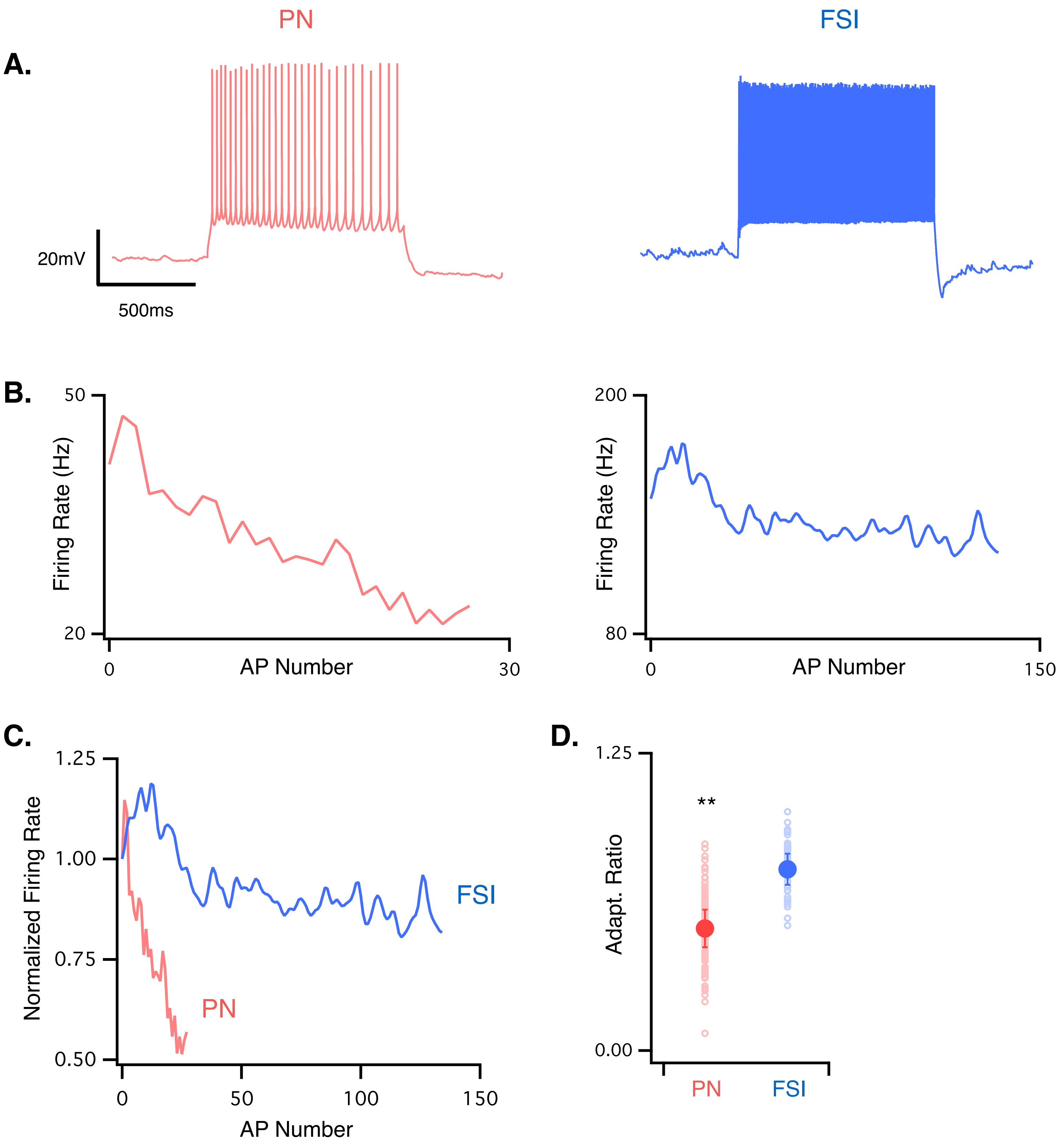

Supplementary Figure 1. FSIs express less spike-frequency adaptation than PNs. **A.** Example traces from a PN (red, left) and an FSI (blue, right) evoked by a 1 second current pulse. **B.** Instantaneous firing rate curves for the traces in *A.* **C.** Same curves as in *B.*, normalized to the 1st firing rate in the trace to illustrate stronger spike-frequency adaptation in the PN. **D.** Mean adaptation ratios (FR_{last} / FR_{first}) for all PNs and FSIs. An adaptation ratio of 1 indicates no change in firing rate over a trace, while an adaptation ratio of 0.5 indicates that the last interspike-interval is twice a long as the first interspike interval. The population FSI adaptation ratio is significantly greater than the PN adaption ratio, reflecting weaker spike-frequency adaptation in FSIs than PNs.

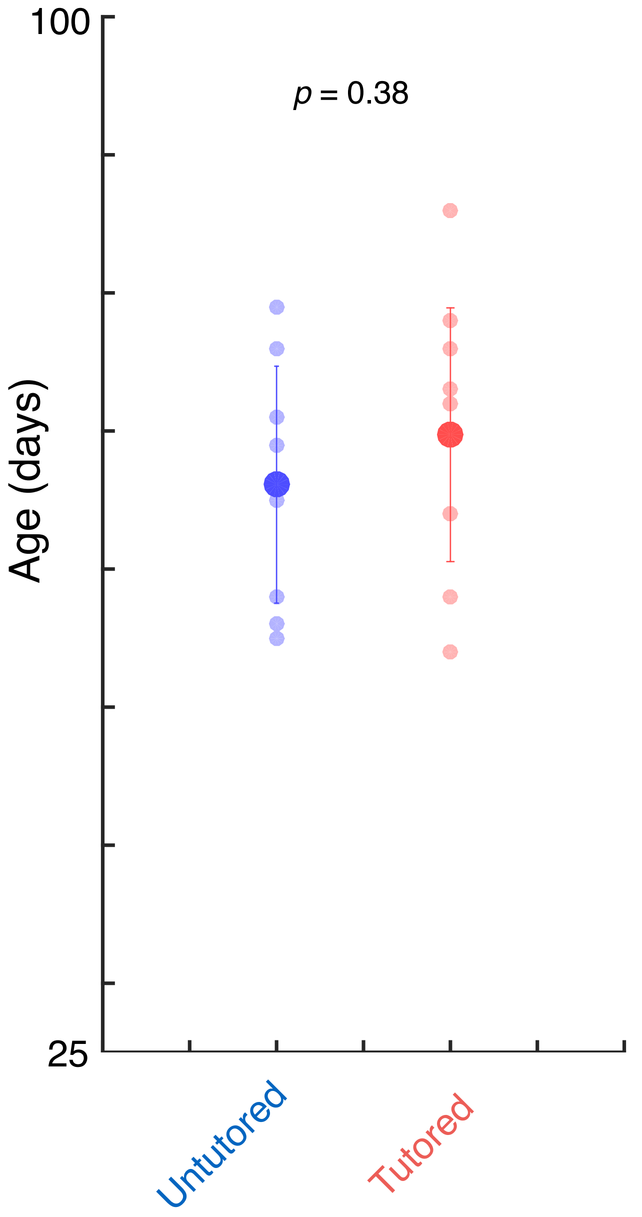

Supplementary Figure 2. Tutored (red) and untutored (blue) birds were not significantly different ages when slice experiments were performed.

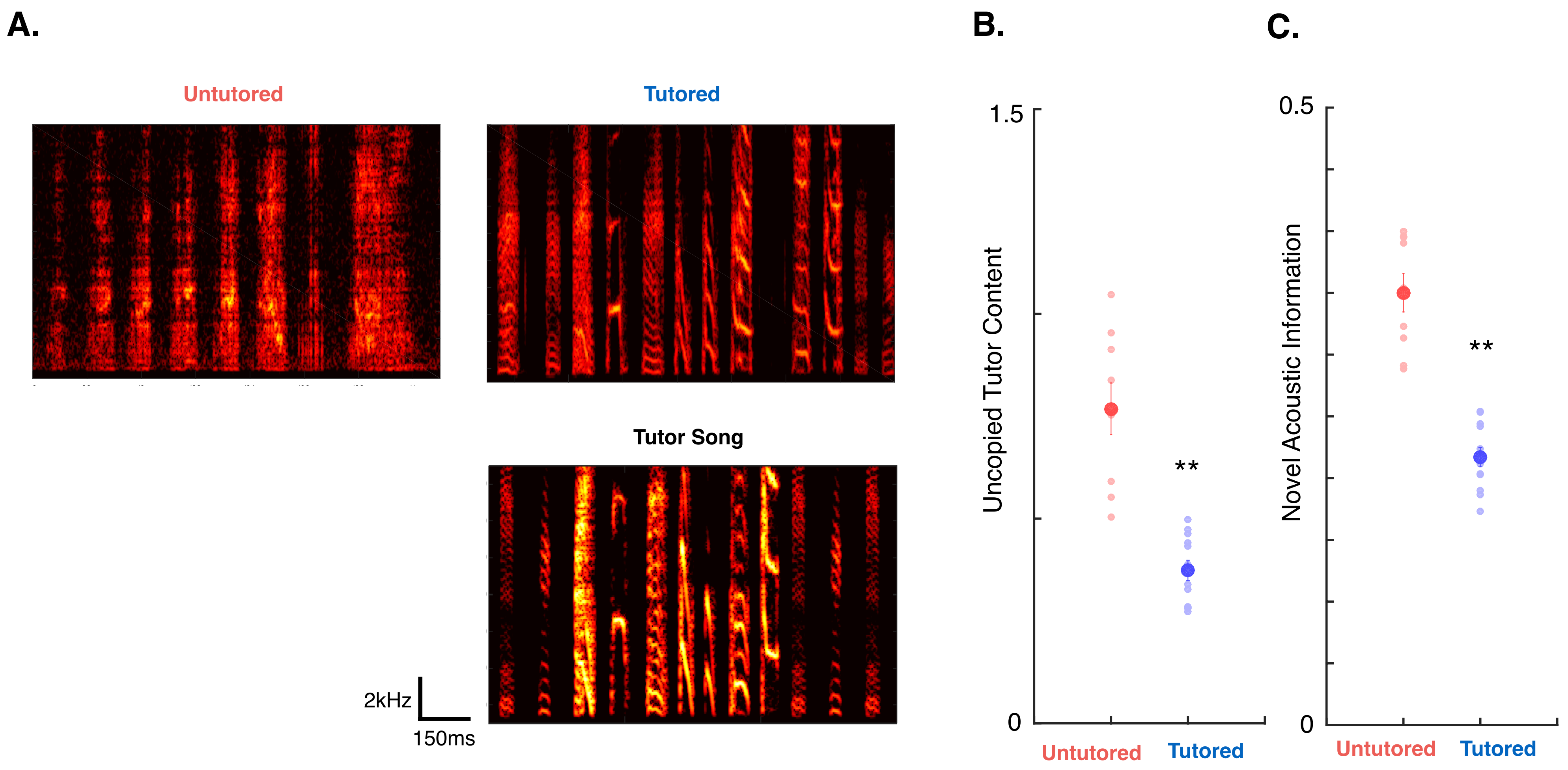

Supplementary Figure 3. Tutored birds learned to produce good copies of the tutor song, while untutored birds of the same age retained unstructured juvenile vocalizations that varied widely across individuals and did not resemble the tutor song. **A.** Song spectrograms from untutored and tutored birds. Each spectrogram is from the bird with the median tutor-similarity score in panel B. **B.** Tutor-similarity values for untutored and tutored birds' songs, quantified by the amount of information in the tutor song that was absent from each birds' song. Smaller values reflect greater similarity to the tutor song: comparison between the tutor song and itself would produce a value of 0. Tutored birds' songs were significantly more similar to the tutor than untutored birds' songs were ($p = 0.3^{-5}$). **C.** Spectral content in untutored and tutored birds' songs that was not present in the tutor song. This measure quantifies novel song features that were produced despite their absence from the tutor stimulus. Untutored birds produced significantly more novel sounds that tutored birds did ($p = 0.1^{-6}$). We did not detect a significant relationship between the uncopied tutor content of of tutored birds' songs and FSI-PN P_C in slices from those birds (linear regression $R^2 = 0.05$, $p = 0.47$).

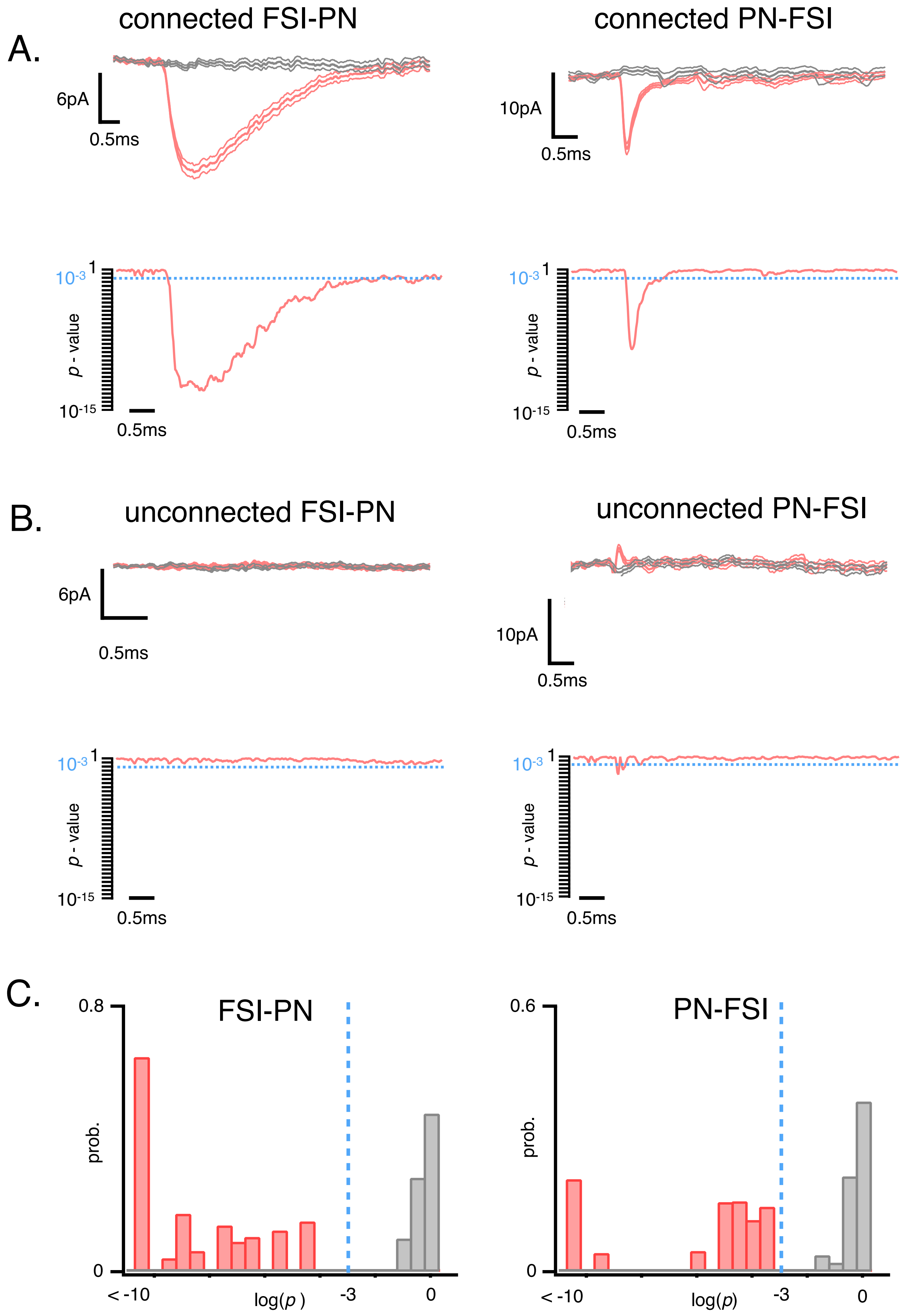

Supplementary Figure 4. Evoked PSCs used to detect synaptic connections in paired recordings were significantly above noise levels in all cases. **A.** Example spike-triggered-average (STA) PSCs from a connected FSI-PN (left) and PN-FSI (right) pair. Red traces are the spike-triggered average IPSC or EPSC and grey traces are null averages triggered on random times in the same traces. Envelopes indicate 95% confidence intervals. Bottom traces indicate the probability of obtaining the STA amplitude from the null distribution at each time point, calculated via *t*-test. We stipulated that a STA must have 0.5ms of consecutive $p < 0.001$ values within 2.5ms of the presynaptic spike to be categorized as a synaptic connection. Blue dotted lines indicate $p = 0.001$. **B.** Same as A, but for FSI-PN and PN-FSI pairs that were classified as unconnected. **C.** Probability distributions of maximum STA *p*-values obtained from all tested FSI-PN and PN-FSI pairs. The *p* threshold of 0.001 is indicated by the blue dotted line. Connected pairs are red and unconnected pairs are grey. All *p* values smaller than 1^{-10} are included in the leftmost bin of each distribution. The unconnected and connected distributions do not overlap in either case.
